# How does digital learning resource accessibility affects math learning anxiety in high school—an empirical analysis based on PISA 2022

**DOI:** 10.3389/fpsyg.2025.1646854

**Published:** 2025-11-14

**Authors:** Guizhen Yang, Cunkuan Wang, Haoran Cui, Yuelong Ming, Zhendong Sun

**Affiliations:** 1 School of Education Science, Ningbo University, Ningbo, China; 2 School of Education Science, Qingdao University, Qingdao, China; 3 Institute of Education, Xiamen University, Xiamen, China

**Keywords:** math learning anxiety, digital learning resources, quality, teacher support, sense of school belonging

## Abstract

**Introduction:**

As math learning anxiety remains a persistent barrier to student achievement and well-being, it is crucial to understand how digital learning environments can alleviate or intensify this issue.

**Methods:**

Drawing on large-scale PISA 2022 survey data from high-school students, this study examines how the quality, usage frequency and manner of use of digital learning resources relate to students’ math learning anxiety, with teacher support and sense of school belonging tested as moderators.

**Results:**

Linear regression analyses reveal that both the quality of digital learning resources and their manner of use are significantly associated with reduced math learning anxiety, whereas usage frequency shows no significant effect. Moderation analyses further indicate that teacher support consistently buffers the impact of all three dimensions of digital resource accessibility on math anxiety, while sense of school belonging only moderates the effects of quality and manner of use.

**Discussion:**

These findings underscore the importance of ensuring high-quality digital resources, fostering supportive teacher–student interactions, and cultivating students’ sense of belonging to reduce math-learning anxiety. Limitations include the cross-sectional design and self-reported data; future longitudinal or experimental studies should test these patterns across contexts.

## 1 Introduction

As digital technologies become increasingly embedded in educational settings, access to digital learning resources has emerged as a key factor influencing students’ learning experiences and outcomes (Kulal et al., 2024; Valiente et al., 2019). In high school mathematics education, digital tools, such as math software, online tutorials, and internet-enabled devices, are often promoted as means to enhance engagement, support individualized learning, and improve performance (Viberg et al., 2023). However, these benefits are not evenly distributed. Students from different socio-economic backgrounds or school contexts may have unequal access to digital learning resources, raising concerns about equity and inclusiveness in the learning process (Beyene et al., 2023).

At the same time, math anxiety, as a form of academic anxiety characterized by feelings of tension, apprehension, or fear when engaging with mathematical tasks, remains a widespread challenge among high school students (Zhang et al., 2019). Math anxiety can negatively affect students’ confidence, motivation, and performance, contributing to long-term disengagement from STEM-related pathways (O'Leary et al., 2017; Zhang et al., 2019). While various psychological and pedagogical factors have been linked to math anxiety, the potential influence of digital access, both in and out of school, has received relatively limited attention in current research.

Given the increasing reliance on digital technologies in classrooms, it is critical to understand how disparities in digital access might contribute to students’ emotional experiences in learning mathematics. Exploring this relationship can help educators and policymakers identify not only material gaps in digital provision, but also hidden emotional and cognitive barriers that hinder effective learning.

To address this issue, this study uses data from the Program for International Student Assessment (PISA) 2022, which includes rich information on students’ access to digital learning resources, their learning environments, and self-reported levels of math anxiety. By analyzing this large-scale international dataset, the study investigates the relationship between digital access and math anxiety among high school students across varied contexts.

This research contributes to the growing discourse on digital equity by highlighting how access to technology relates not only to academic outcomes, but also to students’ emotional well-being. The findings aim to inform more inclusive strategies in digital education, ensuring that technological advancement in schools supports all learners, cognitively and emotionally.

Departing from the traditional “have-versus-have-not” digital divide discourse, this study proposes a three-dimensional accessibility framework (i.e., resource quality, manner of use, and frequency of use) and examines their distinct links to math learning anxiety. Extant research typically treats teacher support and sense of school belonging as antecedents or outcomes; we advance the literature by positioning both factors as simultaneous moderators that may amplify or buffer the effects of digital resources. Leveraging the latest PISA 2022 cross-national data, this work offers a new theoretical lens and analytical pathway for understanding how digital equity intersects with student psychological well-being across diverse cultural contexts.

## 2 Literature review

### 2.1 Digital learning resources in high school education

The integration of digital learning resources in high school classrooms has expanded rapidly over the past two decades. In mathematics education, High-quality digital learning resources yield a significant positive effect on students’ mathematics achievement (Hedges *g* = 0.28, *p* < 0.001), whereas low-quality or merely digitized textbooks show negligible gains (Hillmayr et al., 2020). These tools are especially valuable in high school, where students encounter more abstract and complex content that benefits from visual, interactive, and personalized learning supports. Research suggests that digital tools, when meaningfully integrated, can improve student engagement and achievement in high school math (Tang et al., 2022).

Beyond cognitive outcomes, digital access also influences high school students’ emotional and psychological experiences. Several studies suggest that students who lack access to digital learning tools may feel frustrated, excluded, or embarrassed, particularly when their peers can complete assignments more easily or participate more fully in class (Selwyn, 2016; Hohlfeld et al., 2017). In high school math, where many students already struggle with self-confidence and anxiety, such feelings can be intensified by technological gaps.

### 2.2 Inequitable access and the digital divide in high schools

Despite the pedagogical potential of digital resources, high school students do not have equal access to them. The digital divide, which refers to differences in access to technology and internet connectivity, remains prominent across socioeconomic lines, particularly between urban and rural schools, and between high- and low-income families (Warschauer and Matuchniak, 2010). In many cases, students attending under-resourced high schools have limited access to functional digital devices, reliable Wi-Fi, or high-quality digital content, which can hinder their participation in digitally supported instruction (Becker and Park, 2011).

These access disparities were brought into sharper focus during the COVID-19 pandemic, when many schools shifted to online or blended learning. Studies found that high school students with inadequate access were more likely to experience learning interruptions, fall behind academically, and disengage from schoolwork (Ashta et al., 2023; Mukuka et al., 2021). Even after returning to in-person instruction, schools that serve disadvantaged populations often continue to struggle with outdated hardware, insufficient IT support, and inconsistent digital infrastructure (Ashta et al., 2023).

### 2.3 Accessibility of digital learning resources in high school education and its measurement indicators

Some studies suggest that digital learning resources in schools can be categorized into high-impact and low-impact types. The former includes intelligent tutoring systems (e.g., Cognitive Tutor) and real-time feedback programs (e.g., Clickers), which have shown the most significant effects on learners. The latter, including simply digitized traditional materials or gamified learning software, have not demonstrated statistically significant advantages for learners (Cheung and Slavin, 2013). Various types of digital learning resources in school settings, including virtual learning environments, communication technologies, and information/resource sharing technologies, can provide primary and secondary school students with broader access to high-quality learning materials, thereby promoting independent and personalized learning (Jewitt et al., 2011). In high school settings, the quality of digital learning resources, including their stability and technological advancement, has a notable impact on students’ cognitive development (Wang and Degol, 2016). At the same time, the usage frequency and manner of using these resources are key factors that determine their effectiveness (Davis, 1989). Therefore, this study estimates accessibility to digital learning resources in high school education through three dimensions: quality of digital learning resources, usage frequency, and manner of use.

### 2.4 Hypotheses development

At the high school level, mathematics comprises numerous abstract and conceptual topics. Students often face difficulties in learning math, which can lead to *math learning anxiety* (Hussein et al., 2022; Kalemkus and Kalemkus, 2022; Videla et al., 2022). Researchers suggest that when math instruction is designed using available digital learning resources, it can stimulate students’ curiosity, making it easier for them to achieve their learning goals (Lai and Cheong, 2022). For instance, two-dimensional mathematical spaces can be visualized as three-dimensional ones, and abstract concepts can be dynamically presented, enhancing students’ experience and understanding of spatial and abstract ideas. Studies have shown that the effective use of ICT in schools can positively influence students’ performance in STEM (Science, Technology, Engineering, and Mathematics) subjects (Villena-Taranilla et al., 2022).

Therefore, this study proposes that if digital learning resources in high school education can adequately support math teaching, students may learn more effectively in a more visual and interactive environment, thus significantly affecting their math learning anxiety. For example, with the help of AR technology, students can interactively experience certain mathematical principles and laws. However, individual differences in technology acceptance may lead to varying outcomes. This can be explained by the Technology Acceptance Model (TAM; Davis, 1989), which posits that an individual’s acceptance of technology depends on its perceived usefulness and ease of use. We argue that these perceptions form the foundation of students’ emotional engagement with digital mathematics resources. Based on this, the following research hypotheses are proposed:

Hypothesis 1a: The quality of *digital learning resources* in high school education significantly affects students’ *math learning anxiety*.

Hypothesis 1b: The usage frequency of *digital learning resources* in high school education significantly affects students’ *math learning anxiety*.

Hypothesis 1c: The manner of using *digital learning resources* in high school education significantly affects students’ *math learning anxiety*.

According to He and Wen (2025) Research on digital literacy has become deeply integrated into the field of teacher education. Having sufficient digital literacy is crucial for effectively conducting digital teaching activities. Hardman (2019) also found that the positive effect of educational digital tools on student performance hinges on how teachers use them. Hillmayr et al. (2020) documented that training for teachers in using digital learning resources plays a critical role in student academic outcomes. A quasi-experimental study conducted by Chao et al. (2024) showed that students’ perceived mathematics *teacher support* significantly and negatively predicts math anxiety through the chain-mediating effect of the teacher–student relationship. Teacher support sets off a chain reaction: it first influences students’ non-cognitive factors, such as learning motivation and engagement, and then impacts cognitive outcomes like academic performance (Yang et al., 2021). Research indicates that teacher support plays a crucial role in the relationship between the use of digital learning resources in high school education and students’ learning experiences, for example, whether teachers have received relevant training, and their experience and skills in using digital tools (Khaerudin et al., 2021). This aligns with the principles of Self-Determination Theory (hereinafter referred to as: SDT) (Ryan and Deci, 2000), whose core premise is that humans possess a natural tendency for self-realization—an inherent, proactive inclination to explore, learn, and master new skills. To foster such intrinsic motivation, it is essential to satisfy the needs for autonomy, competence, and relatedness. Therein, support from authority figures (e.g., teachers) fulfills students’ psychological needs for competence and relatedness, which in turn enhances their motivation and buffers against negative emotions such as anxiety. Accordingly, this study proposes the following hypotheses:

Hypothesis 2a: *Teacher support* moderates the relationship between the quality of *digital learning resources* in high school education and students’ *math learning anxiety*.

Hypothesis 2b: *Teacher support* moderates the relationship between usage frequency and students’ *math learning anxiety*.

Hypothesis 2c: *Teacher support* moderates the relationship between manner of use and students’ *math learning anxiety*.

A student’s *sense of school belonging* has also been shown to positively impact their learning. This sense of belonging is shaped in part by a school’s overall vision for digital resource use, the guidance provided to students and teachers, and the level of technical support. Similarly, SDT identifies a sense of relatedness and belonging as a fundamental psychological need (Ryan and Deci, 2000). A supportive school community can provide the emotional security necessary for students to engage with challenging subjects like mathematics without excessive anxiety. Based on this, the following research hypotheses are proposed:

Hypothesis 3a: A student’s *sense of school belonging* moderates the relationship between the quality of *digital learning resources* in high school education and their *math learning anxiety*.

Hypothesis 3b: A student’s *sense of school belonging* moderates the relationship between usage frequency and their *math learning anxiety*.

Hypothesis 3c: A student’s *sense of school belonging* moderates the relationship between the manner of using digital learning resources in high school education and their *math learning anxiety*.

## 3 Methodology

### 3.1 Data source

The data used in this study came from the PISA collected by the Organisation for Economic Co-operation and Development (hereinafter referred to as: OECD) in 2022. PISA is a large-scale international assessment of students’ learning abilities that has been conducted since 2000. It targets 15-year-old students and evaluates their ability to apply knowledge, skills, and problem-solving in reading, mathematics, and science, with assessments conducted every 3 years. To ensure representativeness, PISA uses a two-stage stratified sampling design: first randomly selecting schools, then randomly selecting students within those schools. The participants included 37 OECD member countries and 44 partner countries or economies, such as Singapore, Japan, Korea, Canada, Estonia, and the Netherlands. PISA 2022 includes questions related to students’ internet usage behaviors and schools’ digital infrastructure, making it suitable for addressing the research questions of this study.

### 3.2 Variable description

#### 3.2.1 Math learning anxiety

The dependent variable in this study is math learning anxiety. The math learning anxiety scale in PISA 2022 includes the following items, such as I get very tense when I have to do mathematics homework; I feel helpless when doing mathematics problems; I worry that I will get poor grades in mathematics. Each item is measured on a 4-point Likert scale (Strongly disagree/Disagree/Agree/Strongly agree). Based on these items, the PISA project generates a derived variable called “ANXMAT” representing the final score for math learning anxiety.

#### 3.2.2 Accessibility to digital learning resources in high school education

In PISA 2022, accessibility to digital learning resources in schools is evaluated through three dimensions: quality of digital learning resources, usage frequency, and manner of use.

- Quality of digital learning resources is measured based on students’ agreement with statements about their school’s digital infrastructure (e.g., There are enough digital devices with access to the Internet at my school; The school’s Internet speed is sufficient; Digital devices function properly at my school). This scale consists of nine items (IC172Q01JA–IC172Q09JA), each rated on a 4-point scale (Strongly disagree = 1 to Strongly agree = 4). PISA also provides a derived variable called “ICTQUAL” based on this scale, which is used in this study.
- Usage frequency refers to how often students use digital learning resources (e.g., computers, tablets, educational software) in school. It is measured by asking, “During this school year, how often did you use the following digital resources at school?” Response options range from “Never or almost never” (=1) to “Several times a day” (=5). There are seven items (IC170Q01JA–IC170Q07JA). PISA also recoded this set by marking unavailable resources as 0 and all others as 1, producing a derived variable “ICTSCH,” which is used in this study.
- Manner of use is assessed through questions on how frequently students use digital tools for school-related tasks, such as “Creating multimedia presentations with pictures, sound, or video,” “Report or share your results from your own experiments or investigations,” and “Analyze data that you have collected yourself.” Each of the 10 items (IC174Q01JA–IC174Q10JA) is rated on a 5-point scale ranging from “Never or almost never” (=1) to “Every day or almost every day” (=5). This study adopts the derived variable “ICTENQ” created by the PISA project based on these items.

#### 3.2.3 Teacher support

In PISA 2022, teacher support is measured based on students’ perceived support from teachers during class. A 4-point scale (Every lesson/Most lessons/Some lessons/Never or almost never) is used to assess students’ perceptions of four types of teacher support: holistic support, targeted support, accompanying support, and persistent support.

Teacher support was measured using a 4-point frequency scale with unequal intervals, whereas all other variables are OECD-calibrated continuous plausible-value indices. Accordingly, the Weighted Likelihood Estimate (WLE) scores were retained without further transformation to preserve international comparability. To better handle ordinal categorical data and capture differences between categories, this study employs the Generalized Partial Credit Model (GPCM). After reverse-scoring students’ responses to the four items, a composite teacher support index (TEACHSUP) is constructed using the GPCM. After reverse-scoring students’ responses to the four items, a composite teacher support index (TEACHSUP) is constructed using GPCM. The four items are as follows:

- Holistic support (ST270Q01JA): How often: The teacher shows an interest in every student’s learning.
- Targeted support (ST270Q02JA): How often: The teacher gives extra help when students need it.
- Accompanying support (ST270Q03JA): How often: The teacher helps students with their learning.
- Persistent support (ST270Q04JA): How often: The teacher continues teaching until the students understand.

#### 3.2.4 Sense of school belonging

The sense of school belonging in PISA 2022 is assessed through items such as: (1) I feel like an outsider (or left out of things) at school; (2) I make friends easily at school; (3) I feel like I belong at school; (4) I feel awkward and out of place at school; (5) Other students seem to like me; (6) I feel lonely at school. Students’ self-confidence in learning is also measured using eight items rated on a 4-point confidence scale (Not at all confident / Not very confident / Confident / Very confident). The PISA project team has generated a derived variable ‘BELONG Sense of belonging (WLE)’ based on these eight items, which was used in this study.

#### 3.2.5 Control variables

These indices have been item response theory-calibrated and scaled to weighted likelihood estimates (hereinafter referred to as: WLE) by the OECD, ensuring cross-national comparability. Accordingly, we use the official WLE values without further transformation. All focal constructs—digital learning resource quality, usage frequency, manner of use, math learning anxiety, teacher support, and sense of school belonging—were measured by student self-report and are therefore susceptible to social-desirability bias, heterogeneous item interpretations, and inflated effect sizes. To enhance transparency and reliability, we simultaneously controlled for student gender, grade level, home digital resources, and family background in every model, thereby stripping out student- and school-level confounders that could jointly shape perceptions and anxiety. Family background is measured using the ESCS index (Index of Economic, Social and Cultural Status) provided in the PISA 2022 data, which is a composite measure derived from the highest parental occupational status, highest level of parental education, and availability of family resources. School-level control variables include the proportion of teachers with a master’s degree and the student-teacher ratio.

## 4 Results

### 4.1 Effects of accessibility to digital learning resources, sense of school belonging, and teacher support on math learning anxiety

As shown in Table 1, with math learning anxiety as the dependent variable and gender, grade level, household digital resources, family background, proportion of teachers with a master’s degree, and student-teacher ratio as control variables, we sequentially examined the effects of the quality of digital learning resources, usage frequency, manner of use, sense of school belonging, and teacher support on math learning anxiety. Collinearity diagnostics showed that all predictors’ Variance Inflation Factors (VIFs) ranged from 1.001 to 1.058, well below the conventional cutoff of 5, indicating no serious multicollinearity (see Supplementary Table S1 for detailed results). The Cohen’s *f*^2^ coefficients ranged from 0.036 to 0.048, consistently exceeding the conventional threshold for a small effect (0.02) and approaching the lower bound of a medium effect (0.05). This indicates that, within a large-scale educational survey sample, the predictors account for a non-trivial proportion of unique variance and possess substantive explanatory significance.

**Table 1:** Regression analysis of accessibility to digital learning resources, sense of school belonging, and teacher support on math learning anxiety.

Models		Math learning anxiety											
		Model 1		Model 2		Model 3		Model 4		Model 5		Model 6	
Variables		*β*	*t*	*β*	*t*	*β*	*t*	*β*	*t*	*β*	*t*	*β*	*t*
Control variable	Gender	−0.37	−80.571^***^	−0.374	−81.412^***^	−0.372	−80.679^***^	−0.372	−79.929^***^	−0.346	−75.656^***^	−0.373	−81.282^***^
	Grade	0.017	2.166^*^	0.019	2.406^*^	0.018	2.264^*^	0.020	2.455^*^	0.021	2.609^**^	0.016	2.027^*^
	Family background	−0.126	−55.115^***^	−0.112	−48.279^***^	−0.127	−55.079^***^	−0.120	−50.851^***^	−0.110	−47.786^***^	−0.129	−56.297^***^
	Household digital resources	0	0.191	−0.008	−3.407^***^	0.001	0.542	−0.003	−1.151	0.002	1.068	0.002	0.879
	% of teachers with master’s degree	−0.134	−20.231^***^	−0.135	−20.297^***^	−0.135	−20.151^***^	−0.135	−20.027^***^	−0.114	−17.26^***^	−0.178	−26.620^***^
	Student–teacher ratio	0.001	3.550^***^	0.000	0.627	0.001	3.527^***^	0.001	2.907^**^	0.001	3.005^**^	0.001	3.685^***^
Independent variable	Quality of digital learning resources			−0.127	−56.027^***^								
	Usage frequency					−0.002	−0.763						
	Manner of use							−0.046	−20.103^***^				
Moderators	Sense of school belonging									−0.166	−69.105^***^		
	Teacher support											−0.137	−59.093^***^
*R* ^2^		0.042		0.055		0.043		0.044		0.061		0.057	
Δ*R*^2^		0.042		0.040		0.043		0.041		0.034		0.045	
*F*		1752.185^***^		1960.412^***^		1501.383^***^		1522.932^***^		2206.921^***^		2021.422^***^	
Cohen’s *f*^2^		0.044		0.042		0.045		0.043		0.036		0.048	

^*^, ^**^, ^***^ indicate statistical significance at the 0.05, 0.01, and 0.001 levels, respectively. Model 1 includes control variables only. Models 2–4 estimate the main effects of quality, usage frequency, or manner of use, respectively, while controlling for covariates. Models 5–6 examine the unique effects of sense of school belonging and teacher support, with all controls held constant.

Model 2 shows that the quality of digital learning resources has a significant negative association with math learning anxiety (*β* = −0.127, *p* < 0.001), supporting Hypothesis 1a. Model 3 indicates that usage frequency does not have a significant negative effect on math learning anxiety (*β* = −0.002, *p* > 0.05), so Hypothesis 1b is not supported. Model 4 shows that the manner of use shows a significant negative association with math learning anxiety. (*β* = −0.046, *p* < 0.001), supporting Hypothesis 1c. Model 5 indicates that sense of school belonging has a significant negative effect on math learning anxiety (*β* = −0.166, *p* < 0.001). And finally, Model 6 shows that teacher support has a significant negative effect on math learning anxiety (*β* = −0.137, *p* < 0.001).

### 4.2 Regression analysis of the moderating role of teacher support

Interaction terms were constructed between the quality of digital learning resources, usage frequency, and manner of use with teacher support, as shown in Table 2. With math learning anxiety as the dependent variable and gender, grade level, household digital resources, family background, proportion of teachers with a master’s degree, and student-teacher ratio as control variables, we tested the moderating effect of teacher support on the relationship between the three aspects of digital resource accessibility and math learning anxiety. Collinearity diagnostics revealed that all predictors had VIF values between 1.001 and 1.065, which are far below the conventional threshold of 5, suggesting that multicollinearity was not a concern (see Supplementary Table S2 for details). The Cohen’s *f*^2^ values ranged from 0.062 to 0.071, consistently exceeding the benchmark for a small effect (0.02) and nearing the lower boundary of a medium effect (0.05). These results suggest that, even within a large-scale educational survey context, the predictors contributed a meaningful amount of unique variance and demonstrated notable explanatory power.

**Table 2:** Regression analysis of teacher support as a moderator in the relationship between accessibility to digital learning resources and math learning anxiety.

Models	Math learning anxiety											
	Model 1		Model 2		Model 3		Model 4		Model 5		Model 6	
Variables	*β*	*t*	*β*	*t*	*β*	*t*	*β*	*t*	*β*	*t*	*β*	*t*
Gender	−0.376	−81.762^***^	−0.376	−81.779^***^	−0.375	−81.318^***^	−0.374	−81.214^***^	−0.374	−80.434^***^	−0.374	−80.453^***^
Grade	0.018	2.316^*^	0.018	2.309^*^	0.017	2.179^*^	0.017	2.148^*^	0.019	2.392^*^	0.019	2.399^*^
Family background	−0.117	−50.326^***^	−0.117	−50.261^***^	−0.129	−56.17^***^	−0.13	−56.213^***^	−0.124	−52.583^***^	−0.124	−52.569^***^
Household digital resources	−0.005	−1.943	−0.004	−1.829	0.004	1.655	0.004	1.399	0.001	0.36	0.001	0.408
% of teachers with master’s degree	−0.172	−25.699^***^	−0.173	−25.75^***^	−0.178	−26.573^***^	−0.178	−26.535^***^	−0.178	−26.217^***^	−0.178	−26.238^***^
Student–teacher ratio	0	1.247	0	1.232	0.001	3.659^***^	0.001	3.715^***^	0.001	3.147^**^	0.001	3.137^**^
Teacher support	−0.117	−49.257^***^	−0.118	−49.497^***^	−0.138	−59.306^***^	−0.141	−60.172^***^	−0.135	−56.933^***^	−0.135	−56.858^***^
Quality of digital learning resources	−0.104	−44.898^***^	−0.104	−44.699^***^								
Usage frequency					−0.002	−1.050	−0.003	−1.491				
Manner of use									−0.034	−15.072^***^	−0.035	−15.118^***^
Quality × teacher support			−0.010	−4.850^***^								
Usage frequency × teacher support							−0.022	−11.627^***^				
Manner of use × teacher support											−0.005	−2.187^*^
*R* ^2^	0.066		0.066		0.057		0.058		0.058		0.058	
Δ*R*^2^	0.066		0.066		0.057		0.058		0.058		0.058	
*F*	2027.198^***^		1804.743^***^		1770.073^***^		1589.328^***^		1751.910^***^		1557.810^***^	
Cohen’s *f*^2^	0.071		0.071		0.060		0.062		0.062		0.063	

^*^, ^**^, ^***^ indicate statistical significance at the 0.05, 0.01, and 0.001 levels, respectively. Models 1–2, 3–4, and 5–6 test the moderating effects of teacher support on the links between quality of digital learning resources, usage frequency, manner of use and math learning anxiety.

**Figure 1:**
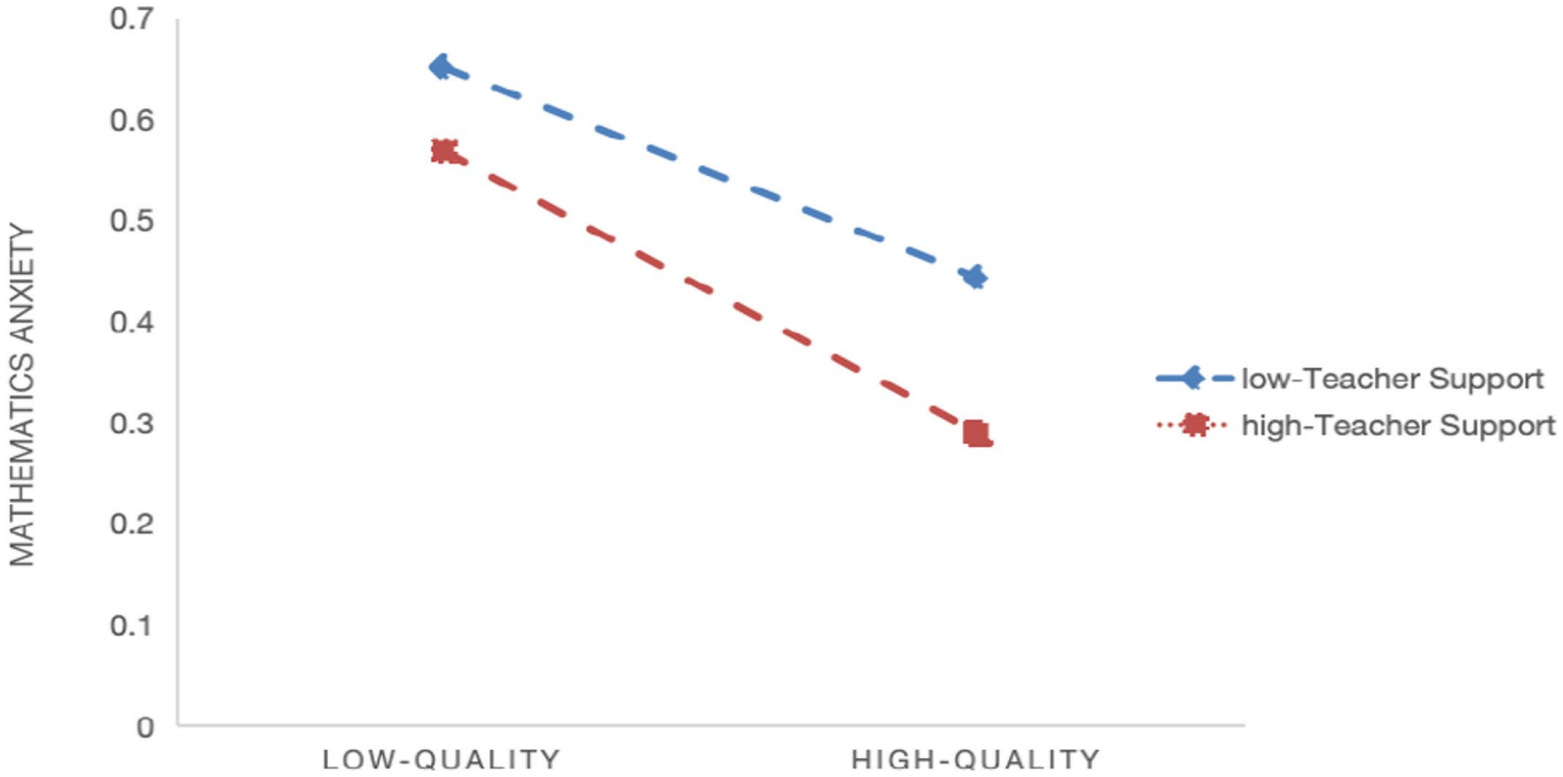
Slope plot of the moderating effect of teacher support on the relationship between quality of digital learning resources and math learning anxiety.

**Figure 2:**
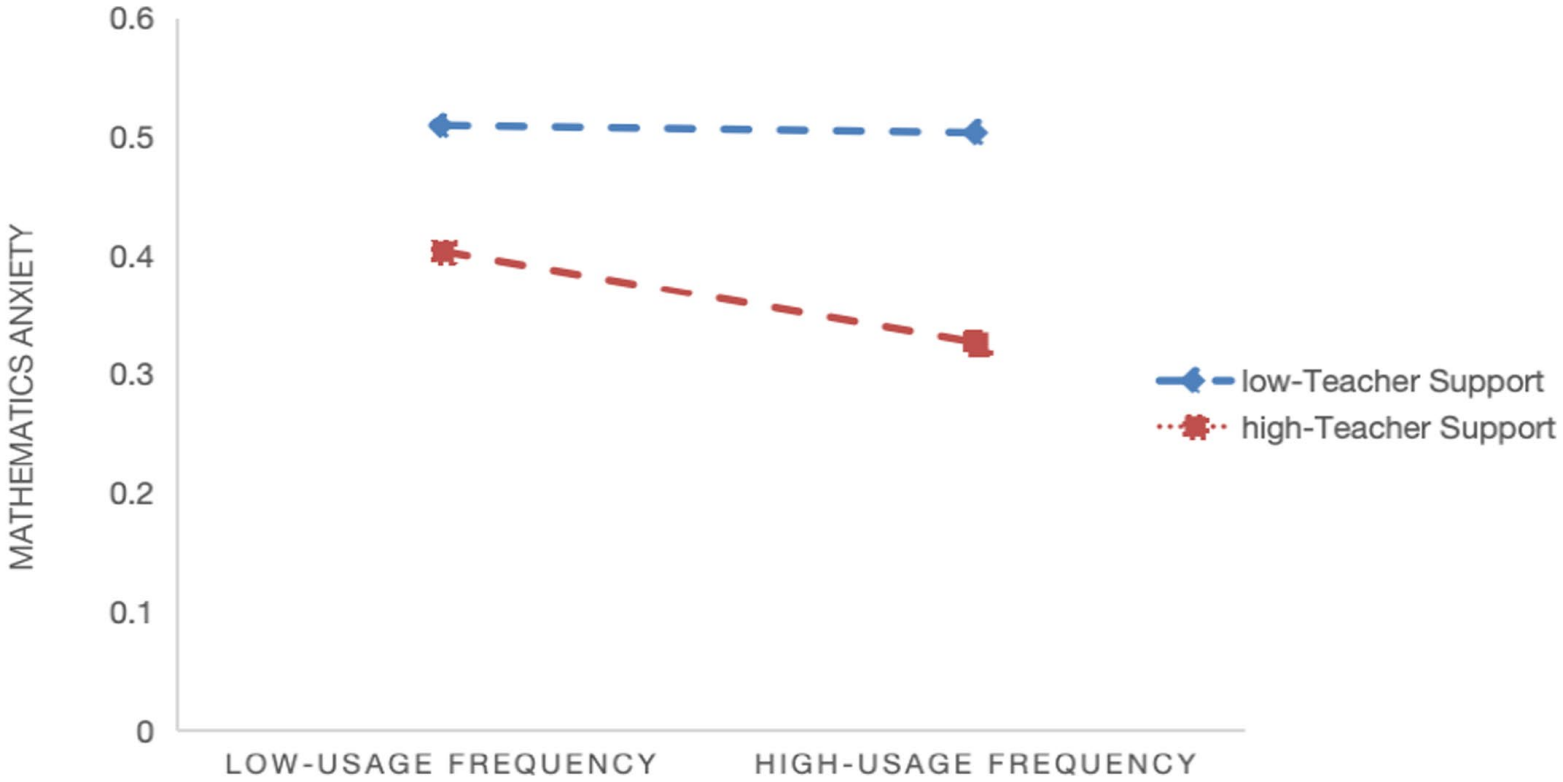
Slope plot of the moderating effect of teacher support on the relationship between usage frequency and math learning anxiety.

**Figure 3:**
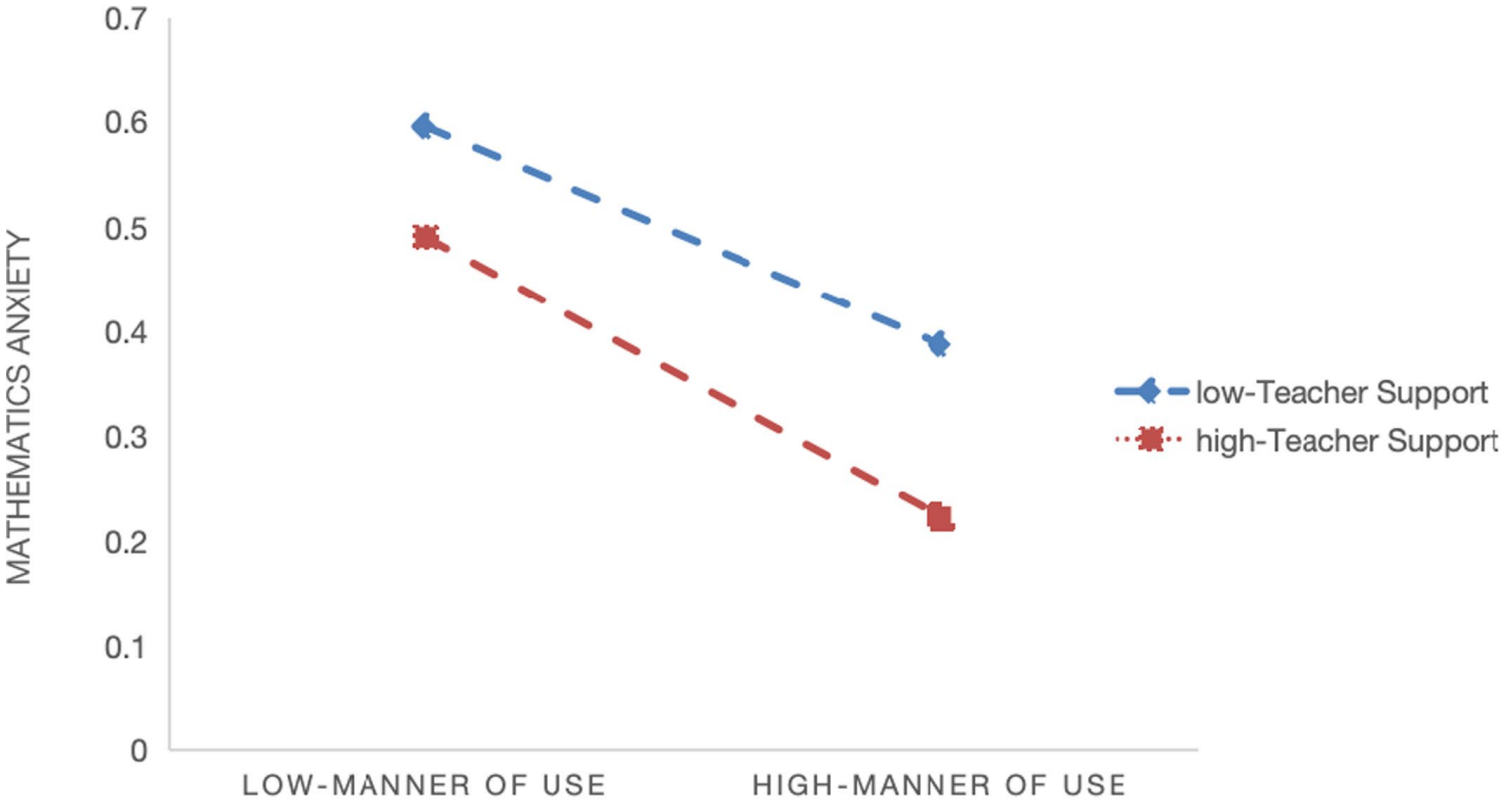
Slope plot of the moderating effect of teacher support on the relationship between manner of use and math learning anxiety.

Model 2 indicates a significant negative moderating effect of teacher support on the relationship between quality of digital learning resources and math learning anxiety (*β* = −0.010, *p* < 0.001), supporting Hypothesis 2a. Figure 1 shows that the negative association between resource quality and math anxiety flattens as teacher support increases. Model 4 shows a significant negative moderating effect of teacher support on the relationship between usage frequency and math learning anxiety (*β* = −0.022, *p* < 0.001), supporting Hypothesis 2b. Figure 2 indicates that the negative association between usage frequency and math anxiety flattens as teacher support increases. And Model 6 indicates a significant negative moderating effect of teacher support on the relationship between manner of use and math learning anxiety (*β* = −0.005, *p* < 0.05), supporting Hypothesis 2c. Figure 3 shows that the negative association between manner of use and math anxiety flattens as teacher support increases.

### 4.3 Regression analysis of the moderating role of sense of school belonging

Interaction terms were constructed between the quality of digital learning resources, usage frequency, and manner of use with sense of school belonging, as shown in Table 3. With math learning anxiety as the dependent variable and gender, grade level, household digital resources, family background, proportion of teachers with a master’s degree, and student-teacher ratio as control variables, we examined the moderating effects of sense of school belonging on the relationships between the three aspects of digital resource accessibility and math learning anxiety. Collinearity diagnostics showed that all predictors’ VIFs ranged from 1.003 to 1.291, well below the conventional cutoff of 5, indicating no serious multicollinearity (see Supplementary Table S3 for detailed results). The Cohen’s *f*^2^ coefficients, ranging from 0.066 to 0.075, were consistently above the small-effect threshold (0.02) and close to the medium-effect benchmark (0.05). This pattern reinforces that, within the large-scale educational dataset, the predictors made statistically and substantively meaningful contributions to explaining the outcome variance.

**Table 3:** Regression analysis of sense of school belonging as a moderator in the relationship between accessibility to digital learning resources and math learning anxiety.

Models	Math learning anxiety											
	Model 1		Model 2		Model 3		Model 4		Model 5		Model 6	
Variables	*β*	*t*	*β*	*t*	*β*	*t*	*β*	*t*	*β*	*t*	*β*	*t*
Gender	−0.352	−76.738^***^	−0.352	−76.721^***^	−0.348	−75.751^***^	−0.348	−75.748^***^	−0.348	−75.022^***^	−0.348	−74.976^***^
Grade	0.022	2.816^**^	0.022	2.818^**^	0.021	2.707^**^	0.021	2.707^**^	0.024	2.973^**^	0.024	2.962^**^
Family background	−0.099	−42.881^***^	−0.099	−42.828^***^	−0.11	−47.756^***^	−0.11	−47.737^***^	−0.105	−44.569^***^	−0.105	−44.466^***^
Household digital resources	−0.005	−2.156^*^	−0.005	−2.169^*^	0.002	0.91	0.002	0.949	0	0.108	0	0.127
% of teachers with master’s degree	−0.117	−17.57^**^	−0.116	−17.564^***^	−0.115	−17.245^***^	−0.115	−17.25^***^	−0.114	−17.024^***^	−0.114	−16.974^***^
Student–teacher ratio	0	0.687	0	0.661	0.001	2.978^**^	0.001	2.966^**^	0.001	2.465^*^	0.001	2.422^*^
Sense of school belonging	−0.149	−61.27^***^	−0.150	−60.142^***^	−0.166	−68.92^***^	−0.166	−68.729^***^	−0.164	−67.157^***^	−0.166	−67.323^***^
Quality of digital learning resources	−0.105	−45.691^***^	−0.105	−45.730^***^								
Usage frequency					−0.001	−0.334	−0.001	−0.471				
Manner of use									−0.035	−15.238^***^	−0.034	−14.976^***^
Quality × sense of school belonging			0.004	2.197^*^								
Usage frequency × sense of school belonging							−0.003	−1.715				
Manner of use × sense of school belonging											0.013	5.959^***^
*R* ^2^	0.070		0.070		0.062		0.062		0.063		0.063	
Δ*R*^2^	0.070		0.070		0.062		0.062		0.063		0.063	
*F*	2196.522^***^		1953.033^***^		1927.625^***^		1713.785^***^		1913.300^***^		1704.913^***^	
Cohen’s *f*^2^	0.075		0.075		0.066		0.066		0.067		0.067	

^*^, ^**^, ^***^ indicate statistical significance at the 0.05, 0.01, and 0.001 levels, respectively. Models 1–2, 3–4, and 5–6 test the moderating effects of sense of schllobelonging on the links between quality of digital learning resources, usage frequency, manner of use and math learning anxiety.

**Figure 4:**
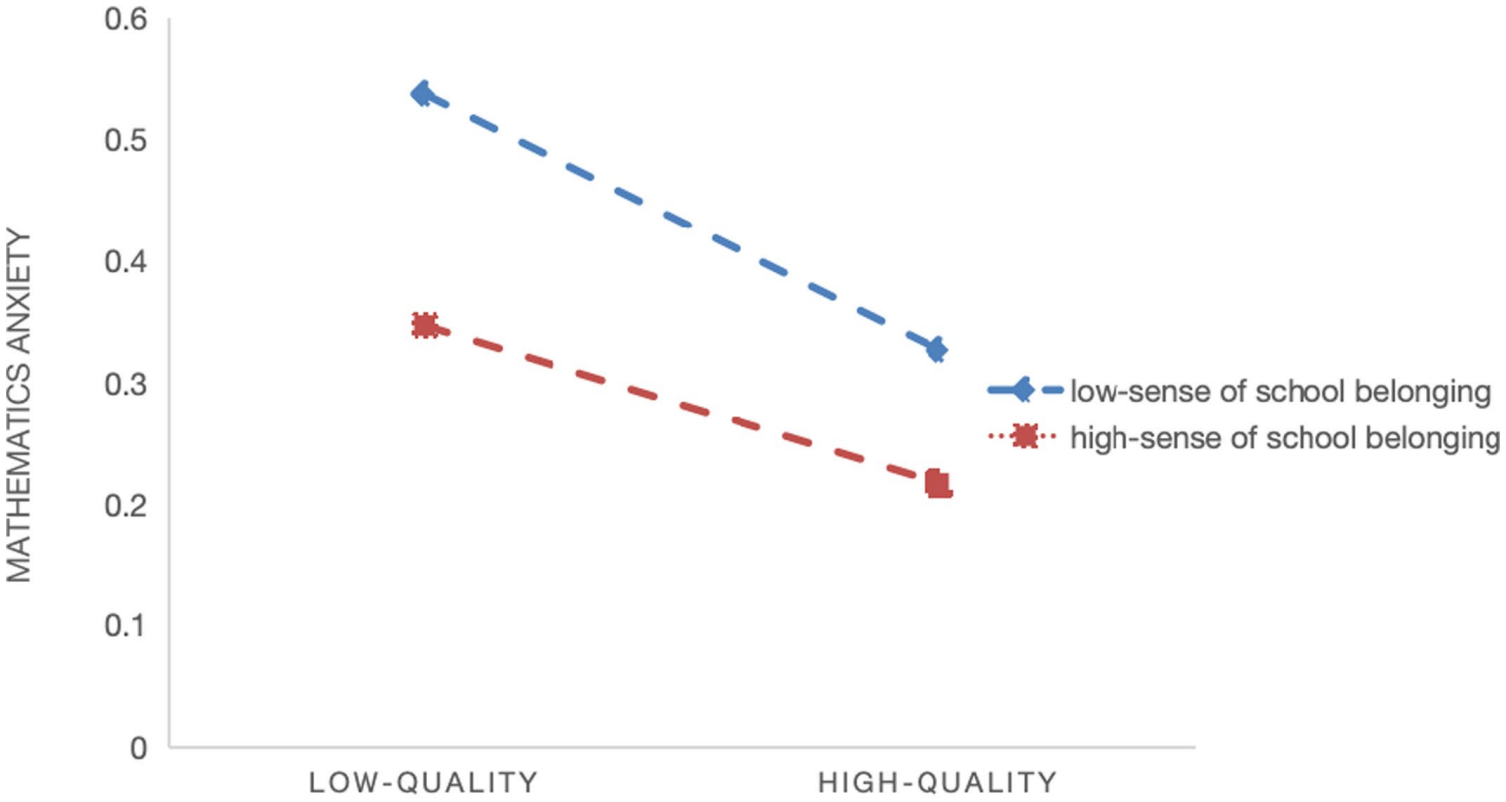
Slope plot of the moderating effect of sense of school belonging on the relationship between quality of digital learning resources and math learning anxiety.

**Figure 5:**
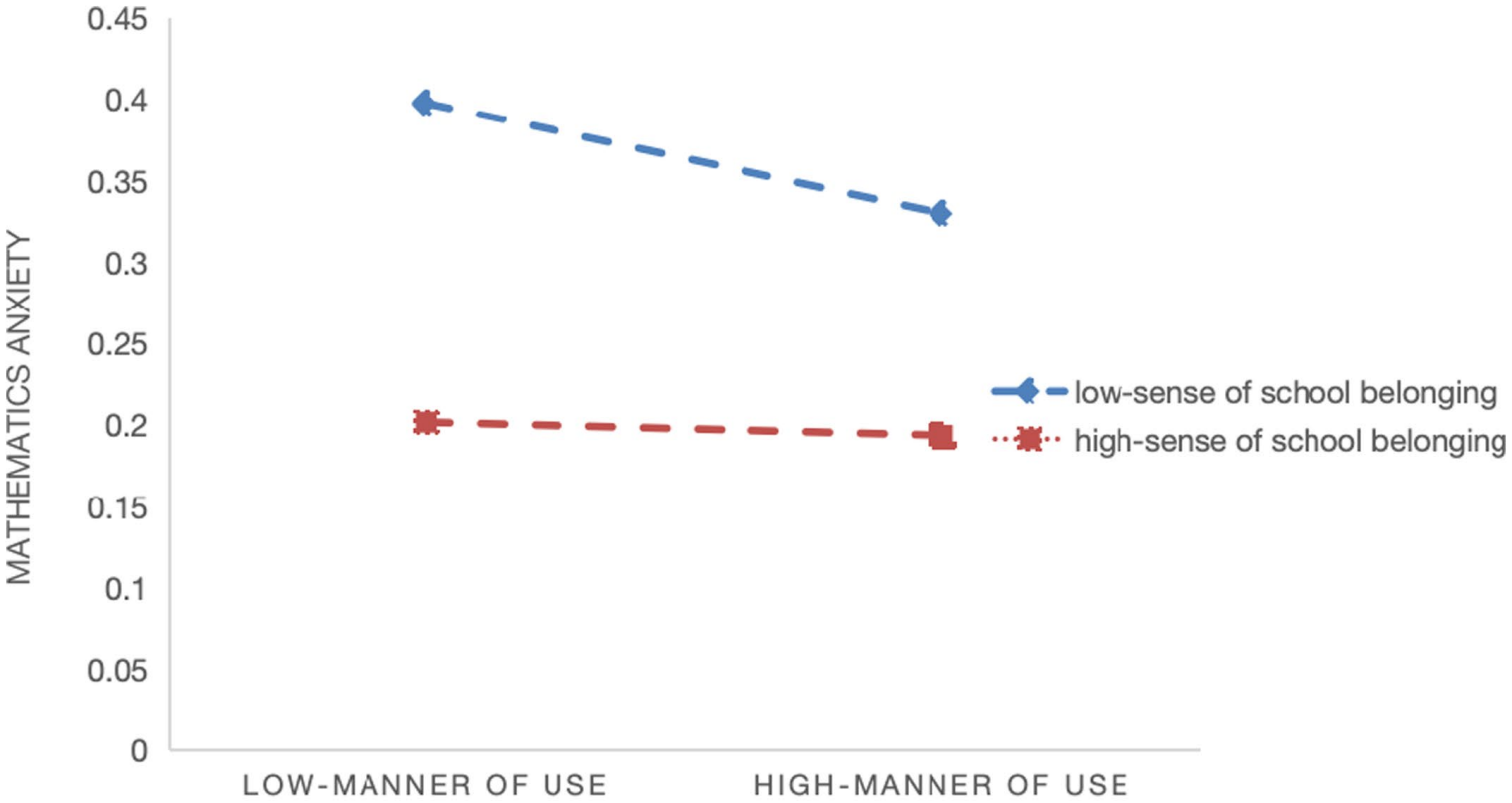
Slope plot of the moderating effect of sense of school belonging on the relationship between manner of use and math learning anxiety.

Model 2 shows a significant positive moderating effect of sense of school belonging on the relationship between quality of digital learning resources and math learning anxiety (*β* = 0.004, *p* < 0.05), supporting Hypothesis 3a. Figure 4 demonstrates that the negative association between digital learning resource quality and math anxiety flattens as students’ sense of school belonging increases. Model 4 indicates no significant moderating effect of sense of school belonging on the relationship between usage frequency and math learning anxiety (*β* = −0.003, *p* > 0.05), so Hypothesis 3b is not supported. Model 6 demonstrates a significant positive moderating effect of sense of school belonging on the relationship between manner of use and math learning anxiety (*β* = 0.013, *p* < 0.001), supporting Hypothesis 3c. Figure 5 shows that the negative association between manner of use and math anxiety flattens as students’ sense of school belonging increases.

### 4.4 Robustness check

To examine the sensitivity of our findings to model specification order, we employed a hierarchical-entry regression framework to test the stability of the main and interaction effects. The entry sequence was as follows:

1. Control block: student- and school-level covariates (gender, grade, family ESCS, home ICT resources, proportion of teachers with a master’s degree, student–teacher ratio);
2. Main-effect block: three dimensions of digital learning resources (quality, usage frequency, manner of use);
3. Moderator block: teacher support and sense of school belonging;
4. Interaction block: product terms between each digital-resource dimension and the two moderators.

Tables 1–3 report Δ*R*^2^, *F*-change statistics, and their significance levels, together with Cohen’s *f*^2^ as an effect-size indicator. The results show that:

1. After entering the main-effect block, the standardized coefficients for “manner of use” and “quality of digital learning resources” remain significantly negative (*p* < 0.001) and exhibit no substantial change in magnitude or direction as subsequent blocks are added.
2. In the interaction block, the negative moderating effects of teacher support and the positive moderating effects of sense of school belonging on “manner of use × belonging” and “quality × belonging” remain consistently robust.
3. Δ*R*^2^ ranges from 0.036 to 0.075, with corresponding Cohen’s *f*^2^ values within the small-to-medium effect range;
4. All F-change statistics are significant at the 0.1‰ level, indicating both statistical and practical contributions to explained variance.

Overall, these findings demonstrate satisfactory stability across alternative model specifications, thereby strengthening the robustness and credibility of the study’s conclusions.

## 5 Discussion

Through linear regression analysis, the study found that both the quality of digital learning resources and the manner of use had significant negative associations with students’ math learning anxiety (*β* = −0.127, *p* < 0.001; *β* = −0.046, *p* < 0.001), supporting Hypotheses 1a and 1c. These results are consistent with previous findings. They suggest that high-quality digital resources and appropriate ways of using them can effectively reduce students’ math learning anxiety. This may be attributed to the fact that high-quality resources provide clearer and more systematic content, helping students better understand and master mathematical concepts, thereby alleviating anxiety during learning (Hillmayr et al., 2020). Furthermore, earlier studies have shown that proper use of digital resources can reduce math anxiety by enhancing students’ motivation and engagement. For example, game-based learning systems and adaptive learning programs not only improve students’ performance but also promote more positive attitudes toward mathematics (Ersozlu, 2024).

In contrast to these findings, the usage frequency of digital resources showed no significant effect on students’ math learning anxiety (*β* = −0.002, *p* > 0.05), indicating that Hypothesis 1b is not supported. This suggests that simply increasing how often digital resources are used does not effectively reduce students’ math learning anxiety. This may relate to how the resources are used. If students are using digital resources passively without truly understanding or internalizing the content, then higher usage frequency may not significantly alleviate anxiety. Student characteristics must also be considered. First, students’ perceived usefulness and perceived ease of use of digital resources help explain this finding. When students perceive digital resources as both beneficial and user-friendly, using them imposes minimal psychological burden, thereby weakening the direct association between usage frequency and anxiety. Second, students’ self-regulatory ability constitutes another important factor. Those with strong self-regulation tend to set clear goals, monitor their progress, and adjust their learning strategies when engaging with digital resources, which gives them a sense of control and direction. As a result, regardless of how frequently they use digital resources, such use remains deliberate and self-managed, substantially reducing the likelihood of learning anxiety. Some prior studies also suggest that usage frequency may influence math anxiety indirectly—such as through learning motivation—but its direct effect is not significant (Li et al., 2023). This implies that without engaging students’ interest, even frequently used digital resources may fail to reduce math anxiety effectively. This suggests that simply increasing students’ exposure to digital devices in the hope of reducing math anxiety is not only futile but may even backfire. Therefore, in educational practice, administrators and teachers should shift their focus to the quality of resources, pedagogical innovation, and teacher–student relationships, ensuring that technology serves educational goals. Only then can digital learning resources truly become a powerful force for alleviating learning anxiety and promoting equity and quality in education.

Moderation analyses using multiple regression revealed that teacher support significantly moderated the relationships between all three dimensions of digital resource accessibility—quality, usage frequency, and manner of use—and students’ math learning anxiety. Specifically, teacher support showed a significant negative moderating effect on the relationship between quality of digital learning resources and math learning anxiety (*β* = −0.010, *p* < 0.001), supporting Hypothesis 2a; a significant negative moderating effect between usage frequency and math learning anxiety (*β* = −0.022, *p* < 0.001), supporting Hypothesis 2b; and a significant negative moderating effect between manner of use and math learning anxiety (*β* = −0.005, *p* < 0.05), supporting Hypothesis 2c. Previous research has also confirmed that teacher support plays a critical moderating role in students’ perceived learning environments and can significantly reduce math anxiety (Chao et al., 2024). Teachers help reduce anxiety by offering emotional support, encouraging participation, and responding to students’ questions in a timely manner, thereby enabling students to better utilize digital resources.

Furthermore, moderation analysis revealed that sense of school belonging positively moderated the relationship between quality of digital learning resources and math learning anxiety (*β* = 0.004, *p* < 0.05), supporting Hypothesis 3a; and also positively moderated the relationship between manner of use and math learning anxiety (*β* = 0.013, *p* < 0.001), supporting Hypothesis 3c. However, the sense of school belonging did not significantly moderate the relationship between usage frequency and math learning anxiety (*β* = −0.003, *p* > 0.05), meaning Hypothesis 3b was not supported. This indicates that the sense of school belonging primarily moderates students’ math learning anxiety through its influence on the quality and manner of use of digital resources. A strong sense of school belonging may enhance students’ confidence and motivation, helping them better adapt to digital learning environments and thereby lowering anxiety (Asanjarani et al., 2024). At first glance, the positive interaction between school belonging and digital resource quality appears counterintuitive because stronger belonging is usually associated with lower anxiety. However, Lam et al. (2015) found that students with higher emotional attachment to their schools also tend to hold stronger expectancy-value beliefs regarding instructional tools. When the quality of educational resources fails to meet these elevated expectations, the resulting “expectation–reality gap” can heighten negative achievement emotions. This interpretation aligns with Chen et al. (2025), who observed that when strong school belonging coincides with high academic pressure, students experience intensified academic anxiety if they perceive instructional support as inadequate. Taken together, these findings suggest that enhancing students’ sense of belonging without concurrently improving the quality of digital infrastructure may unintentionally exacerbate mathematics anxiety among the most engaged learners. Although school belonging plays a notable role in other aspects (e.g., the relationship between quality and anxiety), its moderating role in the relationship between usage frequency and math learning anxiety is not significant. This suggests that usage frequency has a limited direct impact on math anxiety. More specifically, the moderating effect of school belonging may also be limited, possibly because usage frequency is more influenced by external factors such as technical equipment and internet access rather than by students’ sense of belonging (Wang, 2023).

It is important to note that due to the cross-sectional nature of this study, causal inferences cannot be definitively drawn. Future research employing experimental designs or longitudinal data is needed to establish causal pathways.

## 6 Contribution and implications

This study makes several important contributions to the understanding of how digital learning resources, teacher support, and students’ sense of school belonging jointly influence math learning anxiety among high school students in the digital era.

First, it extends existing research by decomposing the accessibility of digital learning resources into three distinct dimensions, including quality, usage frequency, and manner of use, and examining their individual effects on math learning anxiety. The findings reveal that while the quality and manner of use of digital learning resources significantly reduce math learning anxiety, usage frequency alone does not. This underscores the importance of prioritizing resource quality and effective pedagogical integration over mere frequency of use.

Second, this study identifies teacher support as a significant protective factor that not only directly reduces math learning anxiety but also moderates the negative effects of poor-quality digital resources, insufficient usage, and ineffective use. These results highlight the pivotal role of teachers in creating emotionally supportive learning environments that can buffer students from the anxiety often associated with math learning in digital contexts.

Third, the study provides novel insights into the complex role of students’ sense of school belonging. While a strong sense of school belonging is generally associated with lower math anxiety, it unexpectedly amplifies the negative impact of lower-quality resources and ineffective use, suggesting that students who are more emotionally connected to school may also have heightened expectations for their learning experiences. When those expectations are unmet—such as through subpar digital learning environments—these students may experience greater disappointment and, consequently, increased anxiety.

Taken together, these findings offer several practical implications. Educational policymakers and school leaders should: (a) Educational policymakers and school leaders may consider shifting budget priorities from quantity to quality—for example, trialing allocations toward high-impact, interactive software while ensuring stable infrastructure. Given the cross-sectional nature of this study, this suggestion remains exploratory and awaits validation through longitudinal or experimental work. (b) Efforts could be made to explore sustained, practice-based professional-development programs that help teachers integrate digital tools into mathematics pedagogy and balance emotional with instructional support. Yet, as the data are predominantly student-reported, actual teaching practices and their supportive effects need triangulation through classroom observations or teacher interviews. (c) School leaders could pilot a balanced approach that simultaneously fosters students’ sense of belonging and upgrades digital-infrastructure quality, potentially harnessing emotional engagement while mitigating anxiety stemming from unmet expectations. It should be noted, however, that the sample is restricted to Chinese high-school students; thus, the strategy’s applicability in other cultural contexts requires local adaptation and small-scale trials. For Teachers: (a) Focus on pedagogical integration, not frequency of use. Design lessons in which digital tools serve active knowledge creation rather than passive reception. Spark students’ intrinsic interest and, in doing so, reduce the anxiety that often accompanies tool use. (b)Be a supportive guide. Reach out proactively while students are working with technology, offering timely help with both technical glitches and mathematical hurdles. Frame challenges as learning opportunities and explicitly foster a growth mindset, thereby lowering the anxiety tied to mastering new tools.

Overall, this study informs the design of more holistic, student-centered digital education strategies that integrate technological, pedagogical, and social–emotional factors to support students’ learning and well-being in mathematics education.

## 7 Limitations and future research directions

Despite the valuable insights gained from this study, several limitations should be acknowledged. First, this study employed a cross-sectional design, which limits the ability to infer causality between digital learning resource accessibility, teacher support, sense of school belonging, and math learning anxiety. While regression analyses reveal significant associations and moderating effects, longitudinal or experimental designs are needed to validate causal relationships and examine how these effects evolve over time.

Second, the measures of digital learning resources were self-reported by students, which may introduce bias due to subjective perceptions or limited awareness of the actual quality or pedagogical value of the resources used. Future research could incorporate more objective measures of digital resource quality (e.g., expert ratings or metadata analysis) and usage (e.g., learning analytics data).

Third, the study focused exclusively on math learning anxiety among high school students in China, which may limit the generalizability of the findings to other subject areas, age groups, or cultural contexts. Given the subject-specific nature of learning anxiety, future research should explore whether similar patterns exist in other academic domains such as science, language, or social studies.

## 8 Conclusion

This study examined the impact of digital learning resource accessibility in high school settings on senior-high students’ math learning anxiety, and further explored the moderating roles of teacher support and sense of school belonging in these relationships. The present sample consisted solely of 15-year-old senior high school students; therefore, the findings should not be generalized to younger learners or tertiary-level populations. Systematic replication across different educational stages and cultural contexts is essential to evaluate the external validity and boundary conditions of the observed relationships. The key findings are as follows:

1. Linear regression analysis revealed that both the quality of digital learning resources and the manner of use had significant negative associations with students’ math learning anxiety, while usage frequency had no significant effect.
2. Moderation analysis using multiple regression showed that teacher support significantly moderated the relationships between all three dimensions (quality, usage frequency, and manner of use) and students’ math learning anxiety in a negative direction.
3. Multiple regression also showed that sense of school belonging significantly and positively moderated the relationship between quality of digital learning resources and math learning anxiety, and between manner of use and math learning anxiety. However, it did not significantly moderate the relationship between usage frequency and math learning anxiety.

## Data Availability

Publicly available datasets were analyzed in this study. This data can be found here: https://www.oecd.org/en/publications/pisa-2022-results-volume-i_53f23881-en.html.
