# Supplementary material for: How does digital learning resource accessibility affects math learning anxiety in high school—an empirical analysis based on PISA 2022
Source: Front Psychol. 2025 Nov 14;16:1646854. doi: 10.3389/fpsyg.2025.1646854 (PMC12660207; doi:10.3389/fpsyg.2025.1646854)
Supplement: Supplementary file 1 [file Table_1.DOCX]

**Supplementary File**

Table S1. The standard β and VIF for regression analysis of accessibility to digital learning resources, sense of school belonging, and teacher support on math learning anxiety

|  | | Math Learning Anxiety | | | | | | | | | |
| --- | --- | --- | --- | --- | --- | --- | --- | --- | --- | --- | --- |
|  |  | Model 1 | | Model 2 | | Model 3 | | Model 4 | | Model 5 | |
|  |  | *Std. β* | *VIF* | *Std. β* | *VIF* | *Std. β* | VIF | *Std. β* | VIF | *Std. β* | VIF |
| Control  Variable | Gender | -0.164 | 1.001 | -0.163 | 1.001 | -0.163 | 1.001 | -0.151 | 1.007 | -0.163 | 1.001 |
|  | Grade | 0.005 | 1.003 | 0.005 | 1.003 | 0.005 | 1.003 | 0.005 | 1.003 | 0.004 | 1.003 |
|  | Family Background | -0.099 | 1.045 | -0.113 | 1.031 | -0.107 | 1.058 | -0.097 | 1.043 | -0.115 | 1.032 |
|  | Household Digital Resources | -0.007 | 1.013 | 0.001 | 1.289 | -0.002 | 1.018 | 0.002 | 1.009 | 0.002 | 1.009 |
|  | % of Teachers with Master's Degree | -0.041 | 1.028 | -0.041 | 1.028 | -0.041 | 1.029 | -0.035 | 1.03 | -0.054 | 1.041 |
|  | Student-Teacher Ratio | 0.001 | 1.025 | 0.007 | 1.022 | 0.006 | 1.023 | 0.006 | 1.022 | 0.007 | 1.022 |
| Independent Variable | Quality of Digital Learning Resources | -0.114 | 1.021 |  |  |  |  |  |  |  |  |
|  | Usage Frequency |  |  | -0.002 | 1.280 |  |  |  |  |  |  |
|  | Manner of Use |  |  |  |  | -0.042 | 1.035 |  |  |  |  |
| Moderators | Sense of School Belonging |  |  |  |  |  |  | -0.139 | 1.022 |  |  |
|  | Teacher Support |  |  |  |  |  |  |  |  | -0.119 | 1.014 |

Table S2. The standard β and VIF for regression analysis of teacher support as a moderator in the relationship between accessibility to digital learning resources and math learning anxiety

|  | Math Learning Anxiety | | | | | |
| --- | --- | --- | --- | --- | --- | --- |
|  | Model 1 | | Model 2 | | Model 3 | |
|  | *Std. β* | *VIF* | *Std. β* | *VIF* | *Std. β* | *VIF* |
| Gender | -0.165 | 1.001 | -0.164 | 1.001 | -0.164 | 1.001 |
| Grade | 0.005 | 1.003 | 0.004 | 1.003 | 0.005 | 1.003 |
| Family Background | -0.103 | 1.046 | -0.115 | 1.031 | -0.11 | 1.058 |
| Household Digital Resources | -0.004 | 1.014 | 0.003 | 1.291 | 0.001 | 1.018 |
| % of Teachers with Master's Degree | -0.053 | 1.042 | -0.055 | 1.041 | -0.054 | 1.041 |
| Student-Teacher Ratio | 0.003 | 1.024 | 0.008 | 1.022 | 0.006 | 1.022 |
| Teacher Support | -0.103 | 1.065 | -0.123 | 1.024 | -0.117 | 1.022 |
| Quality of Digital Learning Resources | -0.093 | 1.065 |  |  |  |  |
| Usage Frequency |  |  | -0.003 | 1.283 |  |  |
| Manner of Use |  |  |  |  | -0.031 | 1.043 |
| Quality × Teacher Support | -0.010 | 1.010 |  |  |  |  |
| Usage Frequency × Teacher Support |  |  | -0.024 | 1.014 |  |  |
| Manner of Use × Teacher Support |  |  |  |  | -0.004 | 1.002 |

Table S3. The standard β and VIF for regression analysis of sense of school belonging as a moderator in the relationship between accessibility to digital learning resources and math learning anxiety

|  | Math Learning Anxiety | | | | | |
| --- | --- | --- | --- | --- | --- | --- |
|  | Model 1 | | Model 2 | | Model 3 | |
|  | *Std. β* | *VIF* | *Std. β* | *VIF* | *Std. β* | *VIF* |
| Gender | -0.154 | 1.008 | -0.152 | 1.007 | -0.152 | 1.008 |
| Grade | 0.006 | 1.003 | 0.005 | 1.003 | 0.006 | 1.003 |
| Family Background | -0.088 | 1.053 | -0.098 | 1.042 | -0.093 | 1.067 |
| Household Digital Resources | -0.004 | 1.014 | 0.002 | 1.291 | 0 | 1.018 |
| % of Teachers with Master's Degree | -0.036 | 1.03 | -0.035 | 1.03 | -0.035 | 1.03 |
| Student-Teacher Ratio | 0.001 | 1.024 | 0.006 | 1.022 | 0.005 | 1.023 |
| Sense of School Belonging | -0.126 | 1.06 | -0.14 | 1.033 | -0.139 | 1.048 |
| Quality of Digital Learning Resources | -0.094 | 1.052 |  |  |  |  |
| Usage Frequency |  |  | -0.001 | 1.290 |  |  |
| Manner of Use |  |  |  |  | -0.031 | 1.041 |
| Quality × Sense of School Belonging | 0.004 | 1.021 |  |  |  |  |
| Usage Frequency × Sense of School Belonging |  |  | -0.003 | 1.018 |  |  |
| Manner of Use × Sense of School Belonging |  |  |  |  | 0.012 | 1.022 |
